# Intratubular germ cell neoplasms of the testis and bilateral testicular tumors: Clinical significance and management options

**DOI:** 10.4103/0970-1591.60454

**Published:** 2010

**Authors:** Michael C. Risk, Timothy A. Masterson

**Affiliations:** Department of Urology, Indiana University School of Medicine, Indianapolis, IN, USA

**Keywords:** Bilateral testicular cancer, intratubular germ cell neoplasia, testicular germ cell tumor

## Abstract

**Objectives:**

Intratubular germ cell neoplasia (ITGCN) is the precursor lesion for invasive testicular germ cell tumors (TGCTs) of adolescents and young adults. The rising incidence of these tumors has prompted a rigorous investigation of the etiology, diagnosis and management of ITGCN. Bilateral testicular cancer is closely linked with ITGCN, as patients with unilateral testicular cancer are at the highest risk for a future malignancy in the contralateral testicle.

**Methods:**

A literature review directed at ITGCN and bilateral testis cancer was performed using the Medline/PubMed database. Our review focused on the pathogenesis, risk factors, diagnosis and treatment regimens utilized.

**Results:**

Major advances have been made in the understanding of ITGCN over the past 30 years. There is evidence that TGCTs arise from ITGCN, ITGCN is closely related to fetal gonocytes, and that events in pre- and perinatal period may result in abnormal persistence of fetal gonocytes leading to ITGCN and subsequent TGCT. Controversy exists regarding the need to biopsy men at increased risk of TGCT, as well as the best approach to managing patients with known ITGCN. Bilateral testicular cancer has excellent outcomes in the current era of platinum-based chemotherapy.

**Conclusion:**

The optimal management of patients at risk for ITGCN and future TGCT is still a matter of debate. Individualization of management, including biopsy and treatment, should be based on risk factors for TGCT, compliance with potential surveillance, and patient preferences particularly with regard to fertility.

## INTRODUCTION

Testicular cancer is the most common solid tumor in young men, with current estimates in the US being 5.3 cases per 100,000 men.[1] The incidence is rising in the US[1] and worldwide,[2] with the most significant increases seen in countries with the highest incidence.[3] Testicular germ cell tumors (TGCTs) represent the vast majority (90–95%) of malignant tumors of the testis, which are divided according to histologic findings into seminomas and non-seminomas. Both are afflictions of young men, with non-seminomas being more common in the third decade of life, while seminoma is more common in the 4^th^ decade. [2] The rising incidence in these tumors have led to investigations into the pathogenesis of TGCTs, pioneered in part through the initial description of the pre-invasive lesion for TGCTs by Skakkebaek in 1972.[4] This early study described similar lesions in the testis biopsies of two infertile patients who went on to develop TGCTs. While initially termed carcinoma *in situ* (CIS), this same lesion is also referred to in the literature as testicular intraepithelial neoplasia (TIN) and intratubular germ cell neoplasia, unclassified (ITGCN).

Since this seminal work, ITGCN has been found to be the precursor for most TGCTs, with the notable exceptions of pediatric germ cell tumors (yolk sac, mature teratoma) and spermatocytic seminoma. As ITGCN is present years prior to the development of overt cancer,[5] many have sought to improve outcomes in testicular cancer through earlier detection and treatment. Additional studies have begun to shed some light on the molecular events involved in the evolution of ITGCN and TGCT. In this review, we focus on the pathogenesis, risk factors, diagnosis and treatment regimens utilized in the management of ITGCN and bilateral TGCTs.

## PATHOGENESIS

A link between seminoma and non-seminoma was hypothesized prior to the discovery of ITGCN. Seminoma and nonseminoma can co-exist in the same tumor, and, as discussed below, have similar risk factors, suggesting a shared mechanism. Epidemiological studies on TGCTs have demonstrated that incidence trends of seminoma and non- seminoma co-migrate, hinting toward a common etiology.[6] In contrast, there is no correlation of these trends with those seen in pediatric testis tumors, suggesting disparate inciting factors in this group of tumors.[7] Following his initial identification of ITGCN, Skakkebaek found ITGCN involving the residual testis tissue in 17 of 22 orchiectomy specimens performed for TGCT.[8] Other investigators have found ITGCN in as many as 98% of such specimens containing both seminoma and non-seminoma.[9,11]

A link between the ITGCN and TGCTs can also be found in autopsy studies, which identified similar rates of ITGCN in young males to the local incidence of TGCT.[12,13] Subsequent longitudinal studies on patients with ITGCN found that many proceed to develop TGCTs,[14,16]with evidence suggesting a 50% risk in the affected testis by 5 years.[5] Histologic and molecular studies have also shown evidence connecting ITGCN and TGCTs. Placental-like alkaline phosphatase (PLAP) was one of the earliest markers studied, with high circulating levels initially identified in seminoma patients.[17] This led to its investigation as an immunohistochemical marker, where it was found to stain 98% of seminomas, 97% of embryonal carcinomas and 98% of ITGCN specimens with no staining of normal testicular tissues.[18] Since then a number of other immunohistochemical markers have been discovered to share expression in ITGCN and TGCT but not normal testes. These include M2A,[19] 49-3F,[20] TRA-1-60,[21] NANOG,[22,23] AP-2γ,[24] c-kit[25] and Oct ¾.[26] In contrast, markers utilized for pediatric GCTs are unique and do not stain in ITGCN.[27] This is furthered by genetic analyses of ITGCN and its neighboring tumors, which often share chromosomal anomalies suggesting a monoclonal development.[28–30]

Progression of ITGCN to TGCT has been linked to loss of PTEN and p18 expression, as well as induction cyclin E.[31,32] Summersgill and colleagues were able to demonstrate that the gain of chromosome 12p, a common event in TGCTs, allows the ITGCN cells to survive without Sertoli cell interaction.[28,33] Thus, this may be a vital step in the transformation of ITGCN to invasive tumor. Though evidence demonstrates that TGCT does come from ITGCN, the resulting question is the origin of ITGCN itself. Skakkebaek noted, early in his investigation, the morphologic similarities between ITGCN and germ cells at early stages of differentiation as viewed by light or electron microscopy.[34] This hypothesis, which ITGCN stems from gonocytes, was further supported by the expression of many TGCT and ITGCN markers in gonocytes as well,[24,26,35,36] with their disappearance in normal testis by age one year.[37] Additional evidence connecting ITGCN and gonocytes has come through analysis of mRNA expression. Using a differential display PCR technique comparing ITGCN to normal testes, Hoei-Hanson and others were able to demonstrate 28 genes up-regulated in ITGCN, many of which were related to testis development.[38] Microarray analysis was subsequently used to compare the RNA transcriptomes of microdissected samples of ITGCN, gonocytes and other cell types of the fetal gonads.[39] The results demonstrated very similar overall gene expression profiles in ITGCN and gonocytes, with only five genes differentiating the two cell types. Lastly, a number of epigenetic modifications involved in modulating the transcriptional program of primordial germ cells, such as DNA hypomethylation and histone arginine dimethylation, are shared in common between gonocytes and ITGCN.[40,41]

This leads to two potential mechanisms of ITGCN development: either the regression of spermatogonia toward a primordial germ cell phenotype or the abnormal persistence of gonocytes beyond the neonatal period. This is difficult to determine in the lab, in part due to the lack of an animal model for TGCTs. Several epidemiologic studies have investigated the correlation between incidence of cancer development and differences in environmental factors during the time of development and birth. Moller demonstrated decreased testicular cancer incidence in the birth cohort from just before World War II through the end of the war, suggesting an effect prior to or soon after birth.[42] Danish men have the highest risk of testicular cancer, and a recent study demonstrated that risk of testicular cancer is related to county of birth, rather than county of residence at diagnosis.[43] Studies on first and second generation immigrants to Sweden have shown that while first generation immigrants retain TGCT risk similar to their country of origin, the second generation has a risk similar to the Swedish incidence.[44,45]

Additionally, examination of familial testicular cancer risk shows a standardized incidence ratio (SIR) of 3.8-fold in those whose father had TGCT, while brothers have a variable SIR of 6.6 to 10.8 fold which is related to the proximity of their ages. [46] These correlations between birth cohort and testicular cancer risk suggest that the process begins *in utero*, and thus abnormal persistence of gonocytes leading to ITGCN would be commensurate with this. Investigations in the etiologies are ongoing, with many hypotheses relating the role of agents that alter the testicular hormonal microenvironment to this process. [47]

## RISK FACTORS

As ITGCN is the precursor lesion for TGCTs, ITGCN and TGCT have been shown to have similar risk factors. As the diagnosis of ITGCN currently involves testis biopsy, the rates of ITGCN have not been characterized in all groups. The general incidence of ITGCN in two large autopsy series has been found to be 0.43–0.8%, and correlate with the local testicular cancer prevalence.[12,13] Some of the standard risk factors for TGCTs which have not been investigated extensively in ITGCN include Caucasian race[3,48] and family history,[46] while others have been examined through biopsy studies.

One of the greatest risk factors for TGCTs is a personal history of TGCT, with the contralateral testicle being at a 25-fold higher risk in epidemiologic studies.[49] Large series of men with TGCT who underwent biopsy of the contralateral testicle had fairly consistent rates of ITGCN at around 5%.[5,50,51] Some other risk factors for contralateral ITGCN have also been identified through these studies in men with unilateral TGCT. A number of studies have identified testicular atrophy as an increased risk, with 2.5–4.3 fold higher rates of positive biopsies in this subgroup.[51–53]Diagnosis of TGCT prior to age 30 was also associated with increased risk, to nearly eight-fold in one series.[52] Sonographic heterogeneity in the setting of decreased testicular volume was predictive of testicular biopsy outcome in a series of 78 men with unilateral TGCT.[54]

Infertility is another risk factor for development of TGCTs, with a recent database study of 22,562 men suggesting a three-fold increased risk in men with male factor infertility.[55] The initial identification if ITGCN was made in testicular biopsies from infertile men, and early studies by Skakkebaek estimated a 1.1% risk of ITGCN in this setting. [15] More recently a review of biopsies from 453 patients performed for infertility revealed a 2.2% risk of ITGCN, compared with an estimated 0.45% risk in an age- and birth-matched cohort.[56] In line with the epidemiologic study by Walsh, the authors of this investigation found that all patients with a positive biopsy had co-existent severe oligospermia. A retrospective review of 2739 patients who were biopsied during an investigation for infertility over a period of nearly 40 years had only 16 cases of ITGCN.[16] Of note, all 16 patients had testicular atrophy, and 50% of these patients progressed to invasive TGCT within six years.

Cryptorchidism carries a 4.5-6.3-fold increased risk for TGCT in the affected testicle, and this risk is increased further in the setting of bilateral undescended testicles.[49,57] The unaffected testis is at slightly increased risk, [57,58] and early orchiopexy appears to have a protective effect.[59,60] An early biopsy study on 50 men with a history of undescended testicle (UDT) found four patients (8%) with ITGCN,[61] and another small series found one of 17 patients with a history of UDT had ITGCN.[18] In contrast, a larger study of 300 patients with UDT found ITGCN in only five (1.7%),[62] and the various series in contralateral testis to unilateral TGCT suggest no effect of prior UDT on biopsy outcomes.[51,53] Unlike cryptorchidism, the high risk of testicular cancer seen in patients with a variety of disorders of sexual development has been reflective of high rates for ITGCN in many small series.[63,66]

Testicular microlithiasis (TM) has been a point of controversy in the past, as TM is seen in 1.5-5.6% of healthy patients,[67,68] though incidence of TM is much higher in those with TGCT.[69] In a series of 84 patients with incidentally found TM on testicular ultrasound, 63 were contacted five years after the screening study. One of the patients had developed TGCT at 64 months, resulting in an odds ratio of 317 relative to the incidence in that population.[68] Despite this, 98.6% of men with TM did not develop TGCT in the subsequent five years, thus surveillance of these patients is likely of little benefit. TM in the contralateral testis of men with unilateral TGCT was associated with a 28.6- fold increased risk of ITGCN in one series.[70] Infertile men have TM found on ultrasound in 2-20%, and biopsy series in these patients suggest TM is an increased risk of harboring ITGCN especially if it is bilateral or associated with sonographic heterogeneity or atrophy.[67,71,72] Due to this higher incidence of ITGCN in those with TM and other risk factors, surveillance or biopsy has been considered in these patients.

## DIAGNOSIS

ITGCN is diagnosed by testicular biopsy, and has an appearance similar to that of seminoma. The abnormal germ cells are larger than normal spermatogonia, have large hyperchromatic nuclei with prominent nucleoli, and contain abundant cytoplasm with conspicuous cell borders.[73] The tubular basement membrane is often thickened, and the tubules vary from containing adjacent normal Sertoli cells and spermatogonia to complete filling with ITGCN cells.[9] There is usually a heterogeneous distribution of ITGCN with intervening normal tubules throughout the testicle.[74,75] Testicular biopsies should be placed in Bouin's or Stieve's solution, as formalin can make morphologic diagnosis more difficult. As discussed above, a wide variety of markers can be used to identify ITGCN via immunohistochemistry (IHC). The need for IHC was demonstrated in a recent review of 20 cases of TGCT which were preceded by a negative testis biopsy.[76]

Seven cases of ITGCN were discovered by expert review on histology alone, however, an additional four were only found with IHC. The most widely used marker has been PLAP,[77] with more recent incorporation of Oct ¾.[26,78] PLAP has a sensitivity of 83-98%, [17,18] however, Oct 3/4 has reported sensitivity and specificity of 100% in some studies.[26,74,78] Semen analysis for the diagnosis of ITGN has been studied in a number of small trials with a variety of techniques, some of which appear promising; however this is still investigational.[79–81]

### Testicular biopsy

Testicular biopsy for the diagnosis of ITGCN was originally based on simulated biopsies *ex vivo* from four orchiectomy specimens.[82] They found that if 10% of the tubules involved ITGCN, a single 3x3x3mm biopsy containing 30-40 tubules would be sufficient for diagnosis. Large series of testicular biopsies performed on the contralateral testicle with unilateral TGCT have demonstrated the false negative rate to be about 0.5% when performed along with IHC for PLAP.[83] The false negatives were thought to be due to the focal nature of ITGCN,[74] and a number of small series and case reports described TGCT despite prior negative biopsies.[16,83,84] Investigations were done to examine the utility of two-site biopsies, which found in a series of 2318 patients a discordance rate of 31%, and an extra yield of 18%.[85] Even with this approach, subsequent TGCTs in patients with prior negative double biopsy have been described.[86]

Complications reported from testicular biopsy range from 3-20%.[87,88] In a series of 1874 patients who underwent testicular biopsy, 52 had complications with 12 requiring repeat surgery, and one testicle was lost.[88] In this same series, patients were followed with imaging studies which demonstrated evidence of hematoma, edema and vascular injury that resolved in the majority of cases. Decrease in testosterone has been reported following testis biopsy for infertility, with some patients developing hypogonadism; however, these cases often involved a larger number of biopsies and the effect was usually self-limited.[89]

The more important question is which patients would benefit from a testicular biopsy, which is an area of controversy with a wide variety of clinical responses to the same data. The most common scenario in which testicular biopsy is utilized to detect ITGCN is in the contralateral testicle of patients with unilateral TGCT, performed at some centers in Denmark and Germany on all patients with TGCT at the time of orchiectomy. Others perform biopsy in those with TGCT in association with other risk factors, such as testicular atrophy, age < 30 or a history of cryptorchidism. Conversely, physicians in the UK and US have not routinely advocated biopsy of the contralateral testicle. Those who perform biopsies have consistently demonstrated a 5% incidence of CIS in the contralateral testicle;[5,50,51] and in a small series of patients, most develop TGCTs within five years.[5,15,16,34,90] The identification of patients early will allow treatment with radiation, and at least potentially spare their testicular endocrine function in contrast to future orchiectomy (see Treatment). It will also prevent the potential complications of delayed diagnosis of TGCT.[91] Lastly, in those with negative biopsies will be reassured, and potentially can undergo less intense surveillance.

The contrasting viewpoint contends that the 5% rate of ITGCN seen in those studies, while similar to reported rates in Europe,[92,93] is greater than that seen in contemporary series on bilateral TGCT in the US.[94–96] In addition, biopsy has at least a 0.5% false negative rate and many cases of recurrence following radiation have been described, thus strict surveillance is still needed. Radiation, aside from destroying the exocrine function, if effective, also has reported rates of hypogonadism requiring androgen supplementation as high as 40%.[97] Lastly, bilateral testicular cancers in the platinum-based chemotherapy era are associated with good outcomes,[94–96,98] thus survival is purported to be unaffected. Until methods of diagnosis and treatment of ITGCN are improved, or a survival benefit is demonstrated with earlier diagnosis, an informed decision needs to be made based on the data presented and individualized for patient risk factors and priorities.

## TREATMENT

Chemotherapy was initially thought to treat ITGCN in early studies,[99] however, modern series,[100,101] as well as orchiectomy specimens following chemotherapy,[102–105] have proved its lack of efficacy. One study estimated the risk of recurrent ITGCN to be 42% at 10 years.[100] Currently, treatment for ITGCN includes orchiectomy, surveillance and radiation to the affected testicle. Orchiectomy is the main treatment approach in three populations: those with unilateral ITGCN and a contralateral normal testicle; those with an atrophic, poorly functioning testis; and those with oligospermia and ITGCN who are pursuing assisted reproductive techniques. In patients with a solitary testicle, treatment for ITGCN needs to be weighed against the resultant infertility and dependence on exogenous testosterone following orchiectomy.

ITGCN is sensitive to radiation therapy, and this method has been used widely for treatment. Initial treatment regimens consisted of 20 Gy delivered over two weeks (2Gy × 10 doses),[106] and demonstrated effective resolution of ITGCN on repeated biopsies up to two years out in 20 patients. As treatment of ITGCN also involves destruction of the normal spermatogonia, patients who undergo radiation therapy to a solitary testis are rendered infertile. There is evidence that many patients with ITGCN in a solitary testis are infertile even prior to treatment,[107,108] though paternity has been documented[109] and improvement in spermatogenesis has been demonstrated following the removal of unilateral TGCTs.[110] Leydig cell function appears to be affected at 20 Gy as up to 25% require hormone supplementation.[106] This has prompted studies on dose reduction, which had similar effects on endocrine function which was not dose- dependent, and worse treatment outcomes at lower doses.[97] Others have reported less effect on androgen production with lower doses of 13 Gy and 16 Gy.[111,112] Recurrences following radiation therapy have been reported at all dose levels up to 20Gy, though most appear effectively treated at doses of 18-20Gy.

Surveillance remains an option for those with a solitary testicle who wish to preserve fertility and endocrine function. Such patients need to be compliant with regular follow-up, and perhaps more importantly with routine testicular self-examinations. Those who do recur in this setting with tumors less than 3 cm may be candidates for partial orchiectomy, as this has been effective in some studies.[113] As most patients have associated ITGCN, most required adjuvant radiation following orchiectomy, and recurrences in this series were only in patients who did not receive radiation. Partial orchiectomy is still investigational however, and patients should be advised of a high risk for orchiectomy if a tumor recurs in that testis.

## BILATERAL TESTICULAR CANCER

Bilateral testicular cancer has been reported to occur at rates of 1-4% in a variety of series from the US and Europe.[92–96,98,114] In all series, the majority of presentations (62-88%) were metachronous, and median time to development of the second tumor was 50-76 months. The pathology of the second tumor was Stage 1 in 43-90% in these series. Overall, the metachronous patients had better disease free and overall survival than the synchronous patients, with a large SEER database study of 462 patients with bilateral TGCT having 93% 10-year overall survival in the metachronous group compared with 85% in the synchronous group. Single institution studies echoed these results, with most reporting only rare deaths due to TGCT in the modern chemotherapy era.[92,94,95,98] In spite of these outcomes, recurrences were reported as late as 18 years following the first tumor,[95] thus prolonged adherence to surveillance is required.

## CONCLUSIONS

ITGCN has been established at the precursor lesion for development of TGCTs, and has led to extensive insight into the pathogenesis of these tumors. Further work on the potential inciting agents involved during the pre- and peri- natal period can potentially alter the current rising trends in testicular cancer incidence. The diagnosis and management of ITGCN remains a dilemma, and indications for testicular biopsy in ITGCN are controversial. Rather than strict guidelines, biopsy and treatment should be an individualized decision based on both risk factors and patient preferences. Orchiectomy and radiation are the only effective treatments currently to prevent TGCT in the affected testicle, and both can lead to infertility and hypogonadism in the solitary testis. Bilateral testicular tumors occur in fewer than 5% of patients with TGCTs, and outcomes are excellent with current standard therapies. Patients with unilateral TGCT require long-term evaluation for potential metachronous tumors.
